# Diagnostic and prognostic values of cerebrospinal fluid CYFRA 21-1 in patients with leptomeningeal carcinomatosis

**DOI:** 10.18632/oncotarget.18405

**Published:** 2017-06-08

**Authors:** Jae-Won Hyun, Ji Hyun Park, Boo Gil Kang, Eun Young Park, Boram Park, Jungnam Joo, Jong Kuk Kim, Su-Hyun Kim, Jee Hyang Jeong, Hyang Woon Lee, Kee Duk Park, Kyung Gyu Choi, Sang-Hyun Hwang, Ho-Shin Gwak, Ho Jin Kim

**Affiliations:** ^1^ Department of Neurology, National Cancer Center, Goyang, Korea; ^2^ Department of Oncology, Konkuk University of Medical College, Seoul, Korea; ^3^ Department of Laboratory Medicine, National Cancer Center, Goyang, Korea; ^4^ Biometric Research Branch, National Cancer Center, Goyang, Korea; ^5^ Department of Neurology, Dong-A University College of Medicine, Busan, Korea; ^6^ Department of Neurology, Ewha Womans University School of Medicine and Ewha Medical Research Institute, Seoul, Korea; ^7^ Graduate School of Cancer Science and Policy, National Cancer Center, Goyang, Korea

**Keywords:** leptomeningeal carcinomatosis, diagnosis, prognosis, CSF, CYFRA 21-1

## Abstract

**Objectives:**

To investigate the diagnostic and prognostic values of cerebrospinal fluid (CSF) CYFRA 21-1 in patients with leptomeningeal carcinomatosis (LMC).

**Methods:**

Concentration of CSF CYFRA 21-1 was detected using electro-chemiluminescent immunoassay. The difference in level of CYFRA 21-1 between 61 patients with LMC and 200 patients with other neurological disease was evaluated, and diagnostic performance of CSF CYFRA 21-1 was investigated. In LMC patients treated with ventriculo-lumbar perfusion (VLP) chemotherapy, prognostic performance of CSF CYFRA 21-1 was evaluated.

**Results:**

The CSF CYFRA 21-1 was significantly higher in LMC patients than that in patients with other neurological diseases (p<0.001). The sensitivity, specificity, accuracy, and positive and negative predictive values were 80.3%, 95.0%, 91.6%, 83.1%, and 94.1% for CSF CYFRA 21-1, and 65.6%, 100%, 92.0%, 100%, and 90.5% for CSF cytology, respectively. The use of high CSF CYFRA 21-1 and/or positive CSF cytology findings resulted in an increased sensitivity of 85.3%, without compromising specificity. LMC patients with high CSF CYFRA 21-1 were more frequently accompanied by positive CSF cytology results than those with low CSF CYFRA 21-1. The median overall survival was longer in LMC patients with low CSF CYFRA 21-1 than in those with high CSF CYFRA 21-1 (p=0.031). During VLP chemotherapy, the clinical responses were found to be correlated with the biological responses, including the level of CSF CYFRA 21-1 and intracranial pressure.

**Conclusions:**

CSF CYFRA 21-1 might be regarded as an additional diagnostic tool for LMC and a potential significant prognostic biomarker in LMC patients treated with VLP chemotherapy.

## INTRODUCTION

Leptomeningeal carcinomatosis (LMC) is defined as multifocal seeding of metastatic carcinogenic cells in the leptomeninges and cerebrospinal fluid (CSF) in patients with solid tumors [1, 2]. Recently, the incidence of LMC has increased along with improvements in the diagnostic neuroimaging modalities and more effective systemic anti-cancer therapy regimens, which allow longer survival of cancer patients [3, 4]. Moreover, the introduction of new therapeutic strategies, including ventriculo-lumbar perfusion (VLP) chemotherapy and small molecular weight target inhibitors, which decrease the disturbance of the CSF flow or blood brain barrier, has resulted in prolonged survival of patients with LMC [5–10]. Therefore, it has become more important to promptly diagnose, as well as predict the prognosis of, patients with LMC in order to select the patients who will benefit the most from active treatment. However, the sensitivity of the gold standard of LMC diagnosis, detection of malignant cells in the CSF, is currently insufficient to allow an early diagnosis of LMC [1, 2, 4, 11]. Additionally, there is no standardized prognostic marker for patients with LMC.

CYFRA 21-1 is a soluble epithelial cytokeratin 19 fragment belonging to the intermediate filament protein family, which is responsible for the mechanical integrity of the cell and cellular processes [12, 13]. CYFRA 21-1 in serum has been proposed as a diagnostic and prognostic biomarker for several cancers such as lung, head/neck, cervical, and pancreatic cancers [14–21]. However, CYFRA 21-1 in the CSF has not been fully elucidated as a robust diagnostic marker for LMC in a diverse spectrum of cancers. One previous study showed that CSF CYFRA 21-1 could be a diagnostic indicator for LMC of lung cancer; however, the study was based on a small sample size and the primary tumor was restricted to only lung cancer [22]. In more recent studies, CSF CYFRA 21-1 was also introduced as a prognostic marker for overall survival (OS) in patients with LMC in breast cancer treated with conventional intrathecal chemotherapy [23, 24], but further investigation is necessary in patients with LMC treated with VLP chemotherapy. Lastly although monitoring of treatment response with an objective quantitative parameter is crucial to decide individualized therapeutic strategies, currently there is no established standardized tool for treatment assessment in LMC patients treated with VLP chemotherapy.

For these reasons, in the present study, we first aimed to investigate the diagnostic performance of CSF CYFRA 21-1 for LMC in cancer patients and in a large control group comprising patients with other neurological diseases (ONDs). Second, we aimed to evaluate the prognostic value of CSF CYFRA 21-1 for predicting OS and treatment response in patients with LMC treated with VLP chemotherapy.

## RESULTS

### Demographics

In the patients with LMC and OND, the male-to-female ratios were 23:38 and 72:128, and the median ages at sampling were 54 and 40 years, respectively (Table 1). The median KPS at the Ommaya reservoir insertion in patients with LMC was 60. VLP chemotherapy with a 15 ml/h perfusion rate was performed in 37 LMC patients, while a perfusion rate of 20 ml/h was used in 2 LMC patients. Fourteen and 8 LMC patients were treated with intra-ventricular chemotherapy and best supportive care, respectively. The most common primary tumor was lung cancer (n=40, 66%), followed by breast cancer (n=14, 23%) and gastric cancer (n=4, 7%). Histologically, adenocarcinoma was most commonly observed. The control group (OND patients) included non-inflammatory neurological disorders (NIND; n=25) and inflammatory neurological disorders (IND; n=175). In the 175 IND patients, 112 patients were in relapse and 63 were in remission status. Among them, 158 patients were examined before the initiation of acute treatment with intravenous steroid pulse therapy or intravenous immunoglobulin therapy.

**Table 1:** Demographics

	LMC patients (n=61)	Controls (n=200)	p-Value
Gender (male:female ratio)	23:38	72:128	NS
Median age at the CSF sampling (range)	54 (32-79)	40 (13-83)	<0.001
Median KPS at the sampling (range)	60 (30-90)	N/A	N/A
Treatment			
VLP with 15ml/hour perfusion rate	37 (61%)	N/A	N/A
VLP with 20ml/hour perfusion rate	2 (3%)	N/A	N/A
Intra-ventricular chemotherapy	14 (23%)	N/A	N/A
Best supportive care	8 (13%)	N/A	N/A
**Primary tumors in LMC patients**		**OND in controls**	
Lung	**40**	IND	**175**
NSCLC	39	CNS	**153**
Adenocarcinoma	38	NMOSD	67
Large cell neuroendocrine	1	MS	64
SCLC	1	ITM	22
Breast	**14**	PNS	**22**
Invasive ductal	10	AMAN	22
Invasive lobular	4	NIND	**25**
Stomach	**4**	Headache	5
Adenocarcinoma	2	Stroke	5
Signet ring cell	2	Peripheral neuropathy	11
Colon (adenocarcinoma)	**1**	Nutritional deficiency	2
Ovarian (epithelial cell)	**1**	Benign CNS tumor	1
Melanoma	**1**	Motor neuron disease	1

LMC: leptomeningeal carcinomatosis; NS: not significant; CSF: cerebrospinal fluid; SD: standard deviation; KPS: Karnofsky performance status; VLP: ventriculolumbar perfusion; N/A: not applicable; NSCLC: non-small cell lung cancer; SCLC: small cell lung cancer; OND: other neurological diseases; IND: inflammatory neurological diseases; CNS: central nervous system; NMOSD: neuromyelitis optica spectrum disorders; MS: multiple sclerosis; ITM: idiopathic transverse myelitis; PNS: peripheral nervous system; AMAN: acute motor axonal neuropathy; NIND: non-inflammatory neurological diseases.

### Diagnostic value of CSF CYFRA 21-1 for LMC

**Figure 1:**
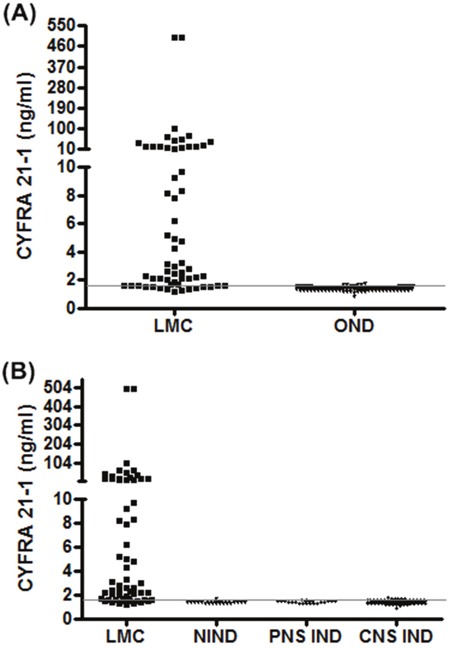
Cerebrospinal fluid (CSF) CYFRA 21-1 levels in patients with leptomeningeal carcinomatosis (LMC) and other neurological diseases (ONDs) **(A)** Patients with LMC and ONDs, **(B)** patients with LMC, non-inflammatory neurological disease (NIND), and central (CNS) and peripheral nervous system (PNS) inflammatory neurological disease (IND). Gray line: diagnostic cut-off value: 1.61 ng/ml.

The CSF CYFRA 21-1 level was significantly higher in LMC patients than in patients with OND (2.94 (1.20-500.00) vs. 1.39 (0.82-1.73) ng/ml, median (range), p<0.001; Figure 1A). When we separately compared the OND patients as NIND (1.37 (1.23-1.62)ng/ml, p<0.001) and IND (including both central [1.40 (0.82-1.73) ng/ml, p<0.001] and peripheral [1.39 (1.23-1.58) ng/ml, p<0.001] nervous system disorders), significant differences were also observed compared with LMC patients (Figure 1B).

The diagnostic cut-off value of CSF CYFRA 21-1 was determined as 1.61 ng/ml by ROC curve analysis, with CSF CYFRA 21-1 over the upper normal limit observed in 49/61 (80.3%) patients. Consequently, a level of ≥1.61 ng/ml was defined as high CSF CYFRA 21-1. The demographics and laboratory characteristics were similar between LMC patients with high and low CSF CYFRA 21-1, whereas CSF cytology-positive findings were more prevalent in LMC patients with high CSF CYFRA 21-1 compared to those with low CSF CYFRA 21-1 (76% vs. 25%, p=0.002; Table 2). The primary tumor of the 49 patients who showed high CSF CYFRA 21-1 included 32 lung (30 adenocarcinoma, 1 large cell endocrine, 1 small cell), 11 breast (8 invasive ductal, 3 invasive lobular), 4 stomach (2 adenocarcinoma, 2 signet ring cell), 1 colon (adenocarcinoma), and 1 ovarian (epithelial cell) cancer. Nine patients with CNS IND (4 neuromyelitis optica spectrum disorder, 4 multiple sclerosis, 1 idiopathic transverse myelitis) and 1 patient with NIND showed high CSF CYFRA 21-1, and 7 of 9 patients with CNS IND were in relapse status before acute treatment.

**Table 2:** Characteristics of LMC patients with high and low CSF CYFRA 21-1

	CYFRA 21-1 ≥1.61 ng/ml(n=49)	CYFRA 21-1<1.61 ng/ml(n=12)	p-Value
Gender (male:female ratio)	19:30	4:8	NS
Median age at the CSF sampling (range)	54 (32-79)	53.5 (35-70)	NS
Median KPS at the sampling	60	60	NS
Laboratory features			
ICP > 200mmH_2_O	20/49 (41%)	5/12 (42%)	NS
CSF cytology (positive)	37/49 (76%)	3/12 (25%)	0.002
CSF protein > 50mg/dl	16/49 (33%)	3/12 (25%)	NS

LMC: leptomeningeal carcinomatosis; CSF: cerebrospinal fluid; 1.61ng/ml: diagnostic cut-off value; NS: not significant; SD: standard deviation; KPS: Karnofsky performance status; ICP: intracranial pressure.

The sensitivity, specificity, accuracy, and positive and negative predictive values were 80.3%, 95.0%, 91.6%, 83.1%, and 94.1% for CSF CYFRA 21-1, and 65.6%, 100%, 92.0%, 100%, and 90.5% for CSF cytology, respectively (Table 3). If we diagnosed patients with LMC who had high CSF CYFRA 21-1 and/or positive CSF cytology, the sensitivity was increased to 85.3% without compromising the specificity. Twelve out of 21 cytology-negative patients showed high CSF CYFRA 21-1.

**Table 3:** Diagnostic performance of the CSF CYFRA 21-1 and cytology

	TP (n)	FP (n)	FN (n)	TN (n)	Sensitivity (%)	Specificity (%)	Accuracy (%)	PPV (%)	NPV (%)
High CYFRA 21-1≥1.61ng/ml (95% CI)	49	10	12	190	80.3 (70.4-90.3)	95.0 (92.0-98.0)	91.6 (88.2-94.9)	83.1 (73.5-92.6)	94.1 (90.8-97.3)
CSF cytology (95% CI)	40	0	21	200	65.6 (53.7-77.5)	100 (100-100)	92.0 (88.7-95.3)	100 (100-100)	90.5 (86.6-94.4)
High CYFRA 21-1 and/or CSF cytology (95% CI)	52	10	9	190	85.3 (76.4-94.2)	95.0 (92.0-98.0)	92.7 (89.6-95.9)	83.9 (74.7-93.0)	95.5 (92.6-98.4)

TP: true positive; FP: false positive; FN: false negative; TN: true negative; PPV: positive predictive value; NPV: negative predictive value; CI: confidence interval.

CSF CYFRA 21-1 was estimated in CSF samples obtained from lumbar and intra-ventricular sites in 32 and 29 patients, respectively. The median value of CSF CYFRA 21-1 was higher in samples obtained from the lumbar site than those from the intra-ventricular site, although the difference did not reach statistical significance (3.9 (1.2-500.0) vs. 2.3 (1.3-101.8) ng/ml, p = 0.402). The proportion of patients with high CSF CYFRA 21-1 did not significantly differ between the two groups (26/32 [81%] vs. 23/29 [79%]).

### Prognostic value of CSF CYFRA 21-1 for LMC patients undergoing VLP chemotherapy

**Figure 2:**
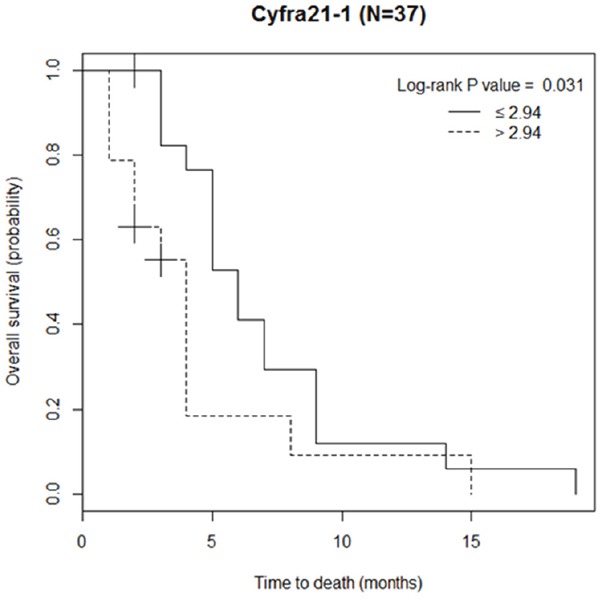
Overall survival of the patients with leptomeningeal carcinomatosis classified by the prognostic cut-off value of cerebrospinal fluid (CSF) CYFRA 21-1 (—— ≤ 2.94 ng/ml, ---- >2.94 ng/ml)

Of the 37 LMC patients treated with VLP chemotherapy with a 15 ml/h perfusion rate, 31 (84%) patients died at the end of the study. The median overall survival for the entire LMC cohort was 5 months (95% confidence interval [CI] 3.0-6.0 months). The prognostic cut-off value was determined as 2.94 ng/ml. The median OS was longer in LMC patients with low CSF CYFRA 21-1 than in those with high CSF CYFRA 21-1. (6 [95% CI: 4.0-9.0] vs. 4 [95% CI: 2.0-4.0] months, p = 0.031; Figure 2). In the univariable analysis (Table 4), CSF CYFRA 21-1 ≤ 2.94 ng/ml, and CSF protein level ≤ 50 mg/dL were found to be significantly associated with favorable OS. The univariable analysis did not reveal any statistical significance of KPS or intracranial pressure at the start of VLP chemotherapy, presence of prior/concurrent radiation therapy, and systemic chemotherapy over 3 different regimens. CSF protein level ≤ 50 mg/dL, continued to be a significant factor in the multivariable analysis.

**Table 4:** Univariable and multivariable Cox regression analysis of prognostic factors of patients with leptomeningeal carcinomatosis who treated with ventriculo-lumbar perfusion chemotherapy

	Univariate (n=37)		Multivariate (n=37)	
**Variables**	**Unadjusted HR (95% CI)**	**p-Value**	**Adjusted HR (95% CI)**	**p-Value**
CSF CYFRA 21-1 level				
≤ 2.94 ng/ml	1			
> 2.94 ng/ml	2.17 (1.03-4.59)	0.042		
CSF protein level				
≤ 50 mg/dL	1			
> 50 mg/dL	3.86 (1.62-9.24)	0.002	3.86 (1.62-9.24)	0.002
KPS at the start of VLP				
< 70	1.69 (0.8-3.56)	0.169		
≥ 70	1			
Intracranial pressure				
< 200 mmH_2_O	1			
≥ 200 mmH_2_O	0.9 (0.43-1.90)	0.786		
Prior/concurrent radiation therapy				
Yes	0.83 (0.39-1.76)	0.628		
No	1			
Prior chemotherapy regimen				
≤3	1			
>3	1.36 (0.66-2.84)	0.407		

HR: hazard ratio; CI: confidence interval; CSF: cerebrospinal fluid; 2.94ng/ml: prognostic cut-off value for leptomeningeal carcinomatosis; KPS: Karnofsky performance status; VLP: ventriculolumbar perfusion chemotherapy.

**Figure 3:**
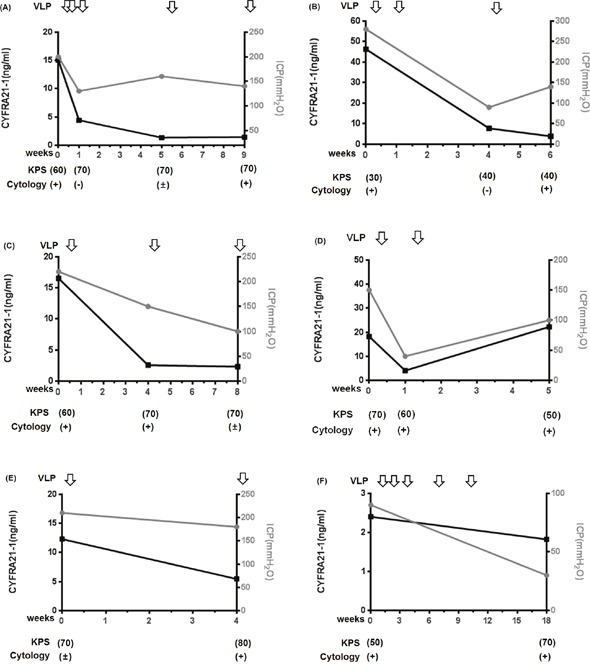
Longitudinal treatment responses during ventriculo-lumbar perfusion (VLP) chemotherapy in 6 patients with leptomeningeal carcinomatosis (A-F) The black squares represent the serial levels of CSF CYFRA 21-1, the gray circles represent the serial levels of intracranial pressure, and the downward-pointing arrows represent VLP chemotherapy treatment. The Karnofsky performance status (KPS) score and cerebrospinal fluid (CSF) cytology results are presented below the graphs. (+) represents positive malignant cells in the CSF, (−) represents negative malignant cells in the CSF, and (±) represents atypical cells in the CSF.

The longitudinal treatment responses during VLP chemotherapy in 6 patients are demonstrated in Figure 3. Five of the 6 patients had lung cancers (adenocarcinomas) while 1 patient had breast cancer (invasive lobular carcinoma). All were treated with VLP chemotherapy with a 15 ml/h perfusion rate. In most patients, decreasing levels of CSF CYFRA 21-1, which corresponded with decreasing ICP and increasing KPS scores, were observed. On the other hand, compared to the CSF CYFRA 21-1, cytological response was more randomly associated with changes in the ICP and KPS scores. Levels of CSF CYFRA 21-1 measured at each time point of assessment showed a positive correlation with ICP (*r_s_* = 0.455) and a negative correlation with the KPS scores (*r_s_* = −0.404). The negative correlation between the individual level of CSF CYFRA 21-1 and KPS score at each assessed time point was maintained when analyzed with ICP as a covariate (pr = −0.496).

## DISCUSSION

In the present study, the level of CSF CYFRA 21-1 was significantly higher in the LMC than OND group, and the combination of high CSF CYFRA 21-1 and/or positive CSF cytology results reduced the number of undiagnosed cases. The median level of CSF CYFRA 21-1 and proportion of high CSF CYFRA 21-1 cases were higher in samples obtained from lumbar subarachnoid space than those in intra-ventricular space, although there was no statistical significance. The LMC patients with high CSF CYFRA 21-1 were more frequently accompanied by abnormal CSF cytology findings than those with low CSF CYFRA 21-1. Furthermore, the median OS in patients with low CSF CYFRA 21-1 was longer than that in patients with high CSF CYFRA 21-1. Clinical responses during VLP chemotherapy showed correlations with the biological responses, including CSF CYFRA 21-1 and ICP.

The diagnosis of LMC has been traditionally made either by the detection of malignant cells in the CSF or contrast enhancement in the subarachnoid space on MRI [1, 2, 4]. However, CSF cytology frequently exhibits false negative results, and it is difficult to differentiate LMC from benign meningitis according to nonspecific meningeal enhancement in MRI [27, 28]. Additionally, CSF cytology result is known to be affected by various factors including CSF volume, which should ideally be >10 ml, and it requires immediate processing of the sample in the laboratory [29]. In contrast, CSF CYFRA 21-1 can be examined with relatively small CSF volume (<100 μl) using stored samples. Furthermore, in this study, we confirmed that a combination of CSF CYFRA 21-1 and CSF cytology as a diagnostic marker increased the diagnostic accuracy in LMC patients. These findings suggest that CSF CYFRA 21-1 may represent an additional diagnostic marker to CSF cytology for LMC, and may help facilitate earlier and more accurate diagnosis of LMC in clinical practice.

A previous study showed both high sensitivity (82.9%) and specificity (97.1%) of CSF CYFRA 21-1, with a high cut-off value (5.5 ng/ml) in LMC patients with lung cancer, as compared to the current study [22]. In the present study, the cut-off value of CSF CYFRA 21-1 was calculated based on a large cohort (n=200) of diverse ONDs, whereas in the previous study, it was calculated based on only 35 patients with benign brain tumors. Hence, this discrepancy is likely due to the small sample size and restricted primary tumor in the previous study. External validation within more diverse control groups, including cancer patients without LMC, patients with primary CNS tumors, and patients with meningitis is necessary to establish a consensus of the optimal diagnostic cut-off value in clinical practice.

Not only a diverse spectrum of diseases in the control group, but also various primary tumors in the LMC group were included in the current study. The primary tumor origin included various organs such as lung, breast, ovary, stomach, and colon. Histologically, adenocarcinoma, large cell endocrine, small cell, signet ring cell, and epithelial cell origin cancers were observed as the primary tumor in LMC patients. As CYFRA 21-1, an epithelial cytokeratin fragment, is known to be useful for the diagnosis of LMC in patients with epithelial cell-originated primary tumors [13], we could observe its significance among such diverse primary tumors. However, further larger restropsective and then prospective investigations should be followed for validating diagnostic value of CSF CYFRA 21-1 in patients with various primary tumors other than carcinoma.

The detection rate of malignant cells in CSF has been reported to be influenced by the applicable sampling site [30], hence we can hypothesize that CSF CYFRA 21-1 might also be affected by the applicable sampling site. In the present study, while no significant difference was observed, the level of CSF CYFRA 21-1 tended to be higher in samples obtained from lumbar subarachnoid sites than those from intra-ventricular sites. Given that CSF is primarily produced by the choroid plexus located in the ventricles;[31] the concentration of CSF biomarkers could be affected by a dilution effect of CSF produced and stored in the ventricle. Therefore, theoretically, CSF CYFRA 21-1 would ideally be measured in CSF samples obtained from the lumbar subarachnoid space.

The median OS was longer in patients with low CSF CYFRA 21-1 than in those with high CSF CYFRA 21-1; however, in the multivariable analysis, CSF CYFRA 21-1 level did not show the statistical significance in predicting OS. Only high CSF protein level, which was identified as a significant prognostic factor in our previous study [11], remained significant in the multivariable analysis. On the other hand, two previous studies that analyzed LMC patients with breast cancer treated with conventional intrathecal chemotherapy showed that CSF CYFRA 21-1 was a significant prognostic factor for OS even in the multivariable analysis using a cut-off value of 4 ng/ml. This discrepancy could be explained by the fact that various types of primary cancer were included in our study, whereas only one type of primary cancer was included in these studies, and by the different methods of chemotherapy used [23, 24].

In a previous study of our institution, the response rate of ICP was the main modality of treatment response assessment in LMC patients treated with VLP chemotherapy [7]. In current study, longitudinal changes of CSF CYFRA 21-1 were associated with the changes of ICP and performance status as well. When we analyzed the data using ICP as a covariate, the negative correlation still remained between the individual level of CSF CYFRA 21-1 and the KPS score. Accordingly, CSF CYFRA 21-1 may be utilized as a quantitative index monitoring treatment responses in LMC patients, whereas CSF cytology is merely qualitative. However, as the CSF CYFRA 21-1 was not tested at regular time intervals in current analysis, further longitudinal evaluations using regular time points are needed. Also, because KPS scores are somewhat nonspecific for evaluation of neurological status, additional parameters should be introduced to more accurately reflect the neurological treatment response in LMC patients.

The retrospective nature and recruitment of LMC patients from a single referral center were methodological limitations of current study that may have resulted in an unintentional selection bias. Additionally, as CSF samples were not obtained at the time of LMC diagnosis but rather collected at Ommaya insertion; the samples might not exactly reflect the LMC status at the time of diagnosis. These altogether warrant further prospective studies with larger sample size and also validation in specific type of primary tumor.

In conclusion, based on the large, as well as diverse, spectrum of control and LMC patients with various types of primary tumors, we suggest CSF CYFRA 21-1 as an additional potential diagnostic indicator for LMC and a prognostic biomarker for patients with LMC treated with VLP chemotherapy. With CSF CYFRA 21-1, we might better detect and predict clinical outcomes of LMC, which would be vital to select the candidates who might most benefit from VLP chemotherapy.

## MATERIALS AND METHODS

Between 2013 and 2016, 61 CSF samples from LMC patients with diverse primary solid tumors, treated at the National Cancer Center in Korea, and reserved available 200 CSF samples from patients with OND (n=200) as the control group, obtained from 3 referral centers, were evaluated for the CYFRA 21-1 level. The diagnosis of LMC was made by positive CSF cytology and/or magnetic resonance imaging (MRI) scans showing LMC. The CSF CYFRA 21-1 levels were measured retrospectively from the reserved CSF samples stored at −80°C. CSF from the intra-ventricular or lumbar subarachnoid space was obtained as part of the preoperative evaluation prior to Ommaya reservoir insertion for VLP chemotherapy or intra-ventricular chemotherapy. VLP chemotherapy was performed with a perfusion rate of 15 ml/h and a daily methotrexate dose of 24 mg in 37 of the 61 LMC patients, who enrolled into an ongoing clinical trial for evaluation of the optimal perfusion rate for VLP chemotherapy. Two patients, who participated in a previous clinical trial [6, 7], underwent VLP chemotherapy with a perfusion rate of 20 ml/h. To evaluate the treatment response, available serial CSF samples from 6 patients with LMC were longitudinally evaluated during VLP chemotherapy.

CSF CYFRA 21-1 concentrations were detected by the Cobas e170 analyzer with Elecsys CYFRA 21-1, which uses an electro-chemiluminescent immunoassay (Roche Diagnostics, Penzberg, Germany) [25]. All tests for CYFRA 21-1 were independently performed by investigators blinded to the study endpoint. First incubation was performed with a 20-μl CSF sample, a biotinylated monoclonal CYFRA 21-1-specific antibody, and a monoclonal CYFRA 21-1 antibody labeled with a ruthenium complex, which formed a sandwich complex. After addition of streptavidin-coated micro-particles, second incubation was performed, during which the complex becomes bound to the micro-particles via interaction between biotin and streptavidin. Subsequently, the mixture was aspirated into the measuring cell where the micro-particles become magnetically captured onto the electrode. Application of a voltage to the electrode and oxidation of [Ru(bpy)_3_]^2+^ in the presence of tripropylamine results in chemiluminescent emission, which was detected by a photomultiplier. Finally, the CYFRA 21-1 concentration was determined using a calibration curve.

The primary outcome for prognosis was OS, defined as the time elapsed from the start of VLP chemotherapy to death. The Karnofsky performance status (KPS) and prior/concurrent radiotherapy and systemic chemotherapy regimens, which could affect the prognosis of patients with LMC, were estimated at the start of VLP chemotherapy. The treatment response was evaluated by the clinical, cytological, and biological responses, defined as improvement of the KPS, disappearing malignant cells in the CSF, and decreasing CSF CYFRA 21-1 levels and intracranial pressure (ICP), respectively. To avoid the immediate effect of VLP drainage on the CSF flow in the measurement of ICP and the dilution effect of VLP drainage in the estimation of the level of CSF CYFRA 21-1, the treatment responses were measured immediately before the next VLP chemotherapy cycle.

This study was approved by the institutional review board committee at the National Cancer Center. A written informed consent was obtained from all patients.

### Statistical analyses

The characteristics of 61 LMC patients and 200 controls were summarized in Table 1. The distributions of categorical variables between groups were compared using the χ2 test or Fisher's exact test. The Mann–Whitney test or Student's t-test was used to analyze differences of continuous variables between groups. Two different cut-off values to dichotomize CSF CYFRA 21-1 were considered. The first is a diagnostic cut-off which was calculated so that the predicted probability (the sum of sensitivity and specificity) becomes a maximum in the receiver operating characteristic (ROC) curve. The characteristics of patients grouped by a diagnostic cut-off of CSF CYFRA 21-1 were summarized in Table 2, and diagnostic performances using this cut-off were presented in Table 3. The second is a prognostic cut-off value which was determined using Contal and O'Quigley method [26]. This method is based on the log-rank test statistic using only a subgroup of patients who were treated with VLP chemotherapy with a 15ml/h perfusion rate (n=37) and using OS as an outcome.

A Cox proportional hazard model was used to investigate the prognostic value of various factors associated with OS, including CSF CYFRA 21-1, protein levels, KPS, ICP, presence of prior/concurrent radiotherapy, and prior systemic chemotherapy over 3 different regimens, which were previously described prognostic factors for LMC [1, 2, 7, 11, 23, 24]. All factors were included in the multivariable model and backward variable selection method with an elimination criterion of p-value > 0.05 was applied. To examine the association between CSF CYFRA21-1 and KPS while removing the effect of ICP, the Spearman partial correlation was calculated. For all analyses, p-value less than 0.05 was considered significant and statistical analyses were performed using SAS version 9.3 (SAS Institute Inc., Cary, NC, USA).
